# Flow diverter treatment for saccular unruptured intracranial aneurysms: A systematic review focussing on study quality and initial outcomes

**DOI:** 10.1093/esj/23969873251370992

**Published:** 2026-01-01

**Authors:** Fabian Wenz, Tamara Wiedemann, Gabriel J E Rinkel, Nima Etminan

**Affiliations:** Department of Neurosurgery, University Hospital Mannheim, Medical Faculty Mannheim, University of Heidelberg, Mannheim, Germany; Department of Neurosurgery, University Hospital Mannheim, Medical Faculty Mannheim, University of Heidelberg, Mannheim, Germany; Department of Neurosurgery, University Hospital Mannheim, Medical Faculty Mannheim, University of Heidelberg, Mannheim, Germany; Department of Neurosurgery, University Hospital Mannheim, Medical Faculty Mannheim, University of Heidelberg, Mannheim, Germany

**Keywords:** Flow-diverting stents, intracranial aneurysm, unruptured, small%

## Abstract

**Introduction:**

Flow-diverting (FD) stents are increasingly used to treat small, unruptured intracranial aneurysms (UIA), but high-quality, unbiased data on initial complications and clinical outcomes were limited in previous literature reviews. We updated the literature review to assess quality, potential bias, complications and short-term outcomes in studies on FD-stents for UIAs.

**Patients and methods:**

We systematically searched PubMed, Embase and Cochrane Library until January 9, 2025 for studies on FD-stents for UIAs. We assessed methodological quality using the methodological index for non-randomised studies (poor: 0–9, moderate: 10–13, good: 14–16), and financial conflicts of interest. The primary outcome was neurological outcome according to a validated outcome scale at 1–3 months after treatment. Secondary outcomes were clinical worsening and complications.

**Results:**

We included 13 studies with 743 patients and 806 UIAs, of which 88.4% (95% CI: 85.7%–91.2%) were <10 mm. All studies were uncontrolled. The methodological quality was poor in six and moderate in seven studies. Financial conflicts of interest were reported in six studies. At 1–3 months after treatment, the proportion of patients were for mRS ⩾1 13.3% (95% CI: 10.0%–16.6%), mRS ⩾2 5.3% (95% CI: 3.2%–7.5%), mRS ⩾3 2.4% (95% CI: 0.1%–3.9%) and neurological worsening 3.1% (95% CI: 1.5%–4.6%). Complications within 3 months occurred in 12.7% (95% CI: 10.3%–15.0%).

**Discussion and conclusion:**

The literature on FD-stents is methodologically weak and potentially biased by financial interests but still shows relevant proportions of complications and post-treatment morbidity. Currently, there are no good data supporting the use of FD-stents for UIAs where standard treatment options are available. Randomised-controlled trials are needed to compare safety, efficacy and durability between FD-stents and coiling or clipping.

## Graphical Abstract

**Graphical abstract:**
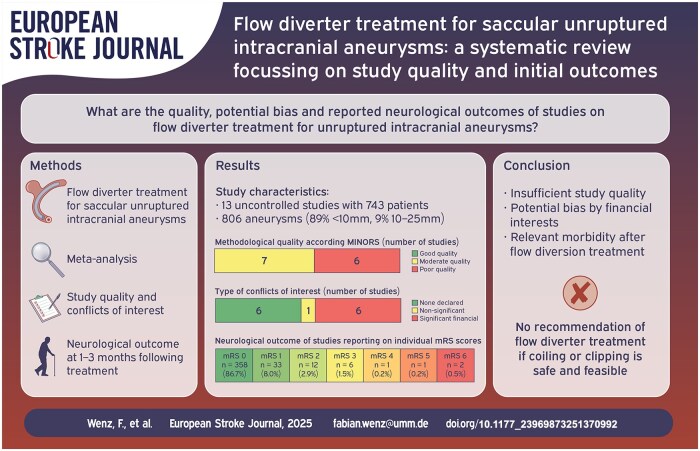

## Introduction

Unruptured intracranial aneurysms (UIA) are prevalent in 3% of the adult population.^1^ Rupture of UIA leads to subarachnoid haemorrhage, which still carries high case-morbidity and -fatality rates.^2^ The current European Stroke Organisation (ESO) guidelines on management of UIA suggest preventive occlusion when the risk of rupture outweighs the risks of the treatment modality that is most effective and safe for that particular aneurysm.^3^ The standard approaches for UIA occlusion are endovascular coiling and surgical clipping, and most UIAs can be treated with one of these two methods.^3–5^ For UIAs with a wide neck, stent-assisted coiling has been introduced, but this procedure is associated with a higher risk of complications than standard coiling,^6^ and proved not superior to standard coiling in a randomised trial comparing the two procedures.^7^ Another device developed to treat aneurysms not amenable for standard coiling or clipping is a flow-diverting (FD) stent. Treatment with FD-stent is also associated with a higher risk of complications than regular coiling,^6^ which may in part be explained by the fact that the FD-stents are used for large and wide UIAs with inherent higher risk of complications than the more usual small UIAs. However, in recent years FD-stents have been increasingly used for non-complex UIA that could be treated with standard coiling or clipping.^8^ Apart from a higher risk of complications, treatment with FD-stents differs in efficacy from standard procedures, because complete occlusion occurs only after several months or not at all.^9^

Because of the high risk of complications associated with treating UIAs with FD-stents, the uncertainty on long term occlusion and the very low quality of the evidence, the ESO guidelines suggest the use of FD-stents for UIAs only when other endovascular or microsurgical options are not feasible.^3^ This statement has subsequently been challenged as already outdated,^10^ referring to a systematic review published after the completion of the guidelines. According to the authors, that systematic review demonstrated overwhelming safety and efficacy of FD-stents.^11^ A critical appraisal of the review, however, showed that it reported only on long-term radiological occlusion rates and complications (aneurysm rupture and late ischaemia) occurring beyond 1 year after treatment. It thus neglected initial complications and did not report on clinical outcome.^12^ Moreover, almost all the studies included in the review were sponsored by industry or written by authors with financial conflicts of interest.

We therefore endeavoured to evaluate the quality of the existing literature, the potential risk of bias from financial conflicts of interest and the short-term clinical outcomes related to the complications of treatment of FD-stents for saccular, non-giant UIAs that can also be treated with standard methods.

## Patients and methods

### Literature research

**Figure 1.**
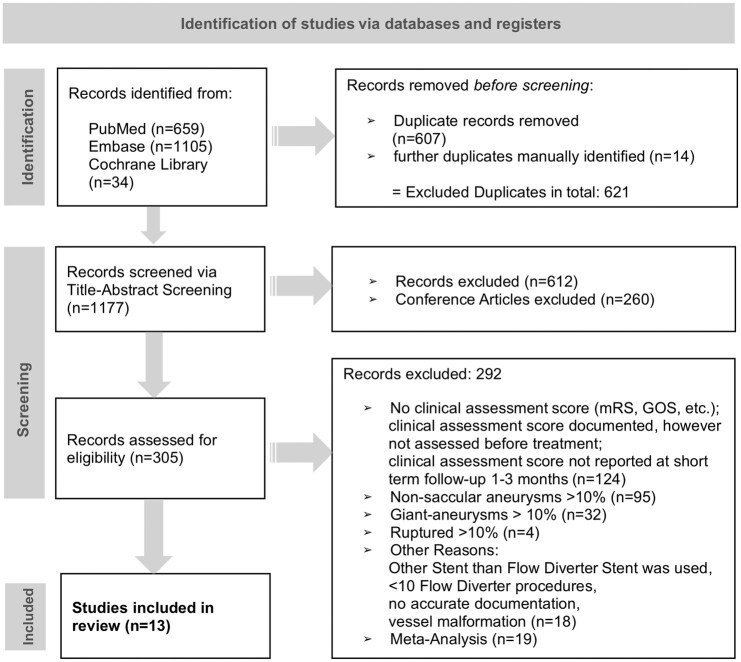
Flowchart. The literature search was performed on 9 January 2025 and 1798 records were identified. After removing 621 duplicates, 1177 studies were screened. Of these, 260 conference abstracts and 612 studies were excluded for not meeting the inclusion criteria. This left 305 reports for further eligibility assessment with full-text review, of which 292 studies were excluded for not meeting the eligibility criteria. The main reason for exclusion was not reporting on clinical outcome of patients <3 months (124 studies). We checked all individual studies that were included in the identified meta-analyses, but all were excluded because they did not meet our eligibility criteria. mRS: modified Rankin Scale, GOS: Glasgow Outcome Scale.

The study was conducted according to PRISMA-guidelines. A comprehensive literature search of PubMed, Embase and the Cochrane Library was conducted with the support of the Mannheim University Library and included studies from inception to 9 January 2025. The search aimed to identify studies reporting on short-term clinical outcomes 1–3 months after treatment of saccular UIAs with FD-stents, using the search terms ((aneurysm* AND (Unruptured OR incidental))) AND (cranial OR intracranial OR cerebral OR brain OR berry OR saccular OR intracerebral) AND (‘flow diver*’ OR ‘pipeline embolization’). The study selection process is shown in Figure 1.

### Eligibility criteria

Studies were included if they met the following criteria: randomised clinical trials or other controlled trials comparing FD-stents with standard treatment (e.g. conventional coiling or surgical clipping) for saccular, non-giant UIAs and reporting short-term neurological outcomes using a validated functional outcome scale. In addition, registry studies and consecutive patient series treated with FD-stents were included if they reported short-term neurological outcomes using a validated scale. Eligible studies had to be focussed on saccular UIA and include more than 10 patients.

Studies were excluded if they met any of the following criteria: studies including more than 10% of fusiform, dissecting or giant aneurysms, studies including more than 10% of patients with ruptured intracranial aneurysms, studies reporting on aneurysms associated with arteriovenous malformations or specific population diseases (e.g. collagen disorders, moyamoya disease) and studies reporting only long-term outcomes without including short-term outcomes for all patients treated with FD-stents during the study period.

### Data collection

Data extraction was performed independently by two reviewers (FW, TW). The extracted data included: (1) basic study information (authors, year of publication, sample size) including conflicts of interest and scores for the Methodological Index for Non-Randomised Studies (MINORS), (2) baseline patient characteristics (age, sex, pre-existing conditions), (3) aneurysm characteristics (size categorised as small for <10 mm, large for 10–25 mm, and giant for >25 mm, location, aneurysm type), (4) clinical outcomes 1–3 months and 12 months after treatment, (5) occlusion grades at 12 months after treatment and (6) complication rates, divided into short term (<3 months) and intermediate (3–12 months after treatment).

### Quality assessment and risk of bias

Quality assessment was performed using the MINORS as all included studies were uncontrolled and the Newcastle-Ottawa Scale could therefore not be used.^13^ The maximum score is 16, and we categorised the level of methodological quality into poor (0–9), moderate (10–13) and good (14–16). Conflicts of interest were defined as significant financial if they involved consulting, proctoring, honoraria or shares from the company producing the FD-stent under study or ownership of this company.

### Clinical outcomes

The primary clinical outcome was short-term neurological outcome at 1–3 months after treatment, assessed using a validated outcome scale. Secondary outcomes were clinical worsening after treatment, which was defined as any worsening of mRS from pre-treatment to post-treatment, clinical outcome and aneurysm occlusion rate at 12 months and all reported treatment-related complications. Complete aneurysm occlusion was defined as O’Kelly-Marotta grading scale D or Raymond-Roy classification I. Complications were categorised as short-term (<3 months) or intermediate (3–12 months). Hypoperfusion complications, included in-stent stenosis, thrombosis or vasospasm) or ischaemic events. Haemorrhagic complications were defined as intracranial bleeding events such as intracerebral haemorrhage or aneurysm rupture, or extracranial bleeding such as groyne haematoma.

### Statistical analysis

Statistical analyses were performed using Python version 3.12.9. Ranked variables were presented as medians with interquartile range, continuous variables as pooled weighted means and categorical variables as simple pooled proportions, each with a corresponding 95% confidence interval (95% CI). If certain characteristics or outcomes were not reported in a study, the study was excluded from that particular analysis. For characteristics or outcomes that could not be pooled due to differences in categorisation between studies (e.g. small aneurysms defined as <15 mm), these studies were excluded from the specific outcome or characteristic analysis.

## Results

### Search results

We included 13 studies with 743 patients and 806 aneurysms.^14–26^ The number of patients per study ranged between 15 and 129. One study reported besides the 3-month clinical outcome also clinical outcome data at 1 year in a separate publication.^27^

Of the 743 patients, 609 (82.0%, 95% CI: 79.2%–84.7%) were female, with a pooled mean age of 57.5 years (range 21–82 years). From studies that reported on additional baseline characteristics 186 of 521 patients (35.7%, 95% CI: 31.6%–39.8%) had a history of smoking, 236 of 575 (41.0%, 95% CI: 37.0%–45.1%) had hypertension and 60 of 291 (20.6%, 95% CI: 16.0%–25.3%) had a history of subarachnoid haemorrhage from another aneurysm.

Of the 806 aneurysms, 759 (94.2%, 95% CI: 92.6%–95.8%) were located in the anterior circulation. For size, 527 intracranial aneurysms could be categorised according to our definition and 466 (88.4%, 95% CI: 85.7%–91.2%) were classified as small (<10 mm) and 47 (8.9%, 95% CI: 6.5%–11.4%) as large (10–25 mm). A detailed overview of patient and aneurysm characteristics is given in Table 1.

**Table 1.** Baseline characteristics. SAH: subarachnoid haemorrhage.

Study	Bhogal et al.^14^	Bhogal et al.^15^	Briganti et al.^16^	Cagnazzo et al.^17^	Castro-Afonso^18^	Döring et al.^19^	López-Callejas^20^	Pikis et al.^21^	Wang et al.^22^	Yakar et al.^23^	Bibi et al.^24^	di Villiers^25^	Dibas et al.^26^
Patients (*n*)	30	26	60	15	21	30	106	33	22	54	116	101	129
Female	18 (60.0%)	15 (57.7%)	48 (80.0%)	10 (66.7%)	17 (81.0%)	23 (76.7%)	97 (91.5%)	27 (81.8%)	18 (81.8%)	44 (81.5%)	93 (80.2%)	89 (88.1%)	110 (85.3%)
Smoking	N/a	N/a	N/a	9 (60.0%)	10 (47.6%)	N/a	11 (10.4%)	25 (75.8%)	N/a	N/a	38 (32.8%)	43 (42.6%)	50 (38.8%)
Hypertension	N/a	N/a	N/a	9 (60%)	17 (81%)	N/a	49 (46.2%)	13 (39.4%)	N/a	25 (46.3%)	27 (23.3%)	44 (43.6%)	52 (40.3%)
Prior SAH	N/a	N/a	N/a	5 (33.3%)	5 (23.8%)	N/a	18 (17.0%)	11 (33.3%)	N/a	N/a	21 (18.1%)	N/a	N/a
Acute SAH	0 (0.0%)	0 (0.0%)	6 (10.0%)	1 (6.7%)	0 (0.0%)	0 (0.0%)	1 (0.9%)	0 (0.0%)	0 (0.0%)	0 (0.0%)	7 (6.0%)	0 (0.0%)	0 (0.0%)
Aneurysms (*n*)	30	27	69	17	27	32	106	38	32	58	120	115	135
Localisation													
Anterior circulation	30 (100%)	27 (100%)	64 (92.8%)	17 (100%)	27 (100%)	25(78.1%)	106 (100%)	35 (92.1%)	31 (96.9%)	58 (100%)	102 (85%)	104 (90.4%)	133 (98.5%)
Posterior circulation	0 (0.0%)	0 (0.0%)	5 (7.2%)	0 (0.0%)	0 (0.0%)	7 (21.9%)	0 (0.0%)	3 (7.9%)	1 (3.1%)	0 (0.0%)	18 (15%)	11 (9.6%)	2 (1.5%)
Size													
Small <10 mm	30 (100%)	27 (100%)	N/a	17 (100%)	27 (100%)	N/a	87 (82.1%)	26 (68.4%)	21 (65.6%)	N/a	N/a	96 (83.5%)	135 (100.0%)
Large 10–25 mm	0 (0.0%)	0 (0.0%)	N/a	0 (0.0%)	0 (0.0%)	N/a	12 (11.3%)	8 (21.1%)	9 (28.1%)	N/a	N/a	18 (15.7%)	0 (0.0%)
Giant >25 mm	0 (0.0%)	0 (0.0%)	2 (2.9%)	0 (0.0%)	0 (0.0%)	0 (0.0%)	7 (6.6%)	1 (2.6%)	2 (6.3%)	1 (1.7%)	1 (0.8%)	1 (0.9%)	0 (0.0%)
Morphology													
Fusiform	0 (0.0%)	0 (0.0%)	2 (2.9%)	0 (0.0%)	0 (0.0%)	0 (0.0%)	1 (0.9%)	2 (5.3%)	0 (0.0%)	3 (5.2%)	3 (2.5%)	0 (0.0%)	0 (0.0%)
Blister-like	0 (0.0%)	0 (0.0%)	0 (0.0%)	0 (0.0%)	0 (0.0%)	0 (0.0%)	1 (0.9%)	1 (2.6%)	0 (0.0%)	2 (3.4%)	1 (0.8%)	0 (0.0%)	0 (0.0%)
Dissecting	0 (0.0%)	0 (0.0%)	0 (0.0%)	0 (0.0%)	0 (0.0%)	0 (0.0%)	1 (0.9%)	0 (0.0%)	0 (0.0%)	0 (0.0%)	0 (0.0%)	0 (0.0%)	0 (0.0%)

### Quality assessment

None of the included studies were randomised or otherwise controlled trials, but all described uncontrolled patient series. Two of the 13 studies had prospective data collection.^18,21^ Six studies had a poor, seven a moderate and none a good methodological quality.

### Risk of bias by financial conflicts of interest

Conflicts of interest were reported in seven studies, of which six involved significant financial conflicts of interest (Table 2). A total of 27 conflicts of interest were reported by 66 authors in these studies. The remaining six studies explicitly declared that there are no conflicts of interest.

**Table 2.** Study quality and risk for bias based on methodological index for non-randomised studies (MINORS) and conflicts of interests (COI).

Study	MINORS	Authors (*n*)	Authors with COI (*n*)	Types of COI
Bhogal et al.^14^	10	5	3	Significant financial: consulting, proctoring, shares
Bhogal et al.^15^	9	6	4	Significant financial: consulting, shares
Briganti et al.^16^	10	6	1	Significant financial: proctoring
Cagnazzo et al.^17^	11	8	4	Significant financial: consulting Non-significant financial: grant
de Castro-Afonso et al.^18^	13	9	0	Non-significant financial: funding from stent-producing manufacturer
Döring et al.^19^	9	12	0	—
López-Callejas et al.^20^	9	11	0	—
Pikis et al.^21^	10	9	0	—
Wang et al.^22^	8	8	0	—
Yakar et al.^23^	9	16	0	—
Bibi et al.^24^	9	11	0	—
de Villiers^25^	10	5	2	significant-financial: consulting
Dibas et al.^26^	12	27	13	significant-financial: consulting, shares non-significant financial: funding from stent-producing manufacturer

### Clinical outcome at 1–3 months after treatment

**Figure 2.**
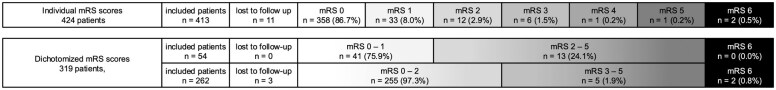
Outcomes reported functional outcomes at 1–3 months grouped as individual and dichotomised mRS scores. mRS: modified Rankin Scale.

All 13 studies used mRS for clinical outcome assessment (summarised in Figure 2), but in only four it was explicitly stated that mRS assessment was done by an observer who was not involved in the treatment of the patient. In two studies it was done by a neuro-radiologist, and in the remaining seven there was no information on who assessed the mRS. Nine studies with 424 patients reported individual mRS scores for neurological outcomes at 1–3 months, of which 11 patients (2.6%, 95% CI: 1.1%–4.1%) were lost to follow-up. Of the 413 patients with available follow-up data at 1–3 months, mRS was ⩾1 in 55 (13.3%, 95% CI: 10.0%–16.6%), ⩾2 in 22 (5.3%, 95% CI: 3.2%–7.5%) and ⩾3 in 10 (2.4%, 95% CI: 0.1%–3.9%) patients, including two reported deaths. For the remaining four studies, only dichotomised mRS were provided and three patients (1.1% 95% CI: 0.0%–2.4%) were lost to follow-up. Three studies reported mRS > 2 in 7 of 262 patients (2.7%, 95% CI: 0.1%–4.6%), including two reported deaths. Another study showed an otherwise defined poor outcome (mRS > 1) in 13 of 54 patients (24.1%, 95% CI: 12.7%–35.5%). Three of the four reported deaths were related to the treatment. A direct comparison between pre- and postinterventional mRS was available in 10 studies, of which 8 were retrospective. These 10 studies described 490 patients, of whom 15 (3.1%, 95% CI: 1.5%–4.6%) had a clinical worsening at short-term follow-up. Based on the comments of one of the reviewers we performed an additional analysis comparing the proportions of patients with mRS ⩾ 3 between studies with and without conflicts of interest. The proportion of patients with mRS ⩾ 3 was 1.2% (95% CI: 0.0%–2.6%) in studies with conflicts of interest and 4.2% (95% CI: 1.2%–7.3%) in those without. We refrained from conducting further analyses (e.g. tests for heterogeneity), due to the small number of studies, lack of comparators and limited methodological quality of the included studies.

### Secondary outcomes

Complete aneurysm occlusion could be extracted in 8 studies and was achieved in 387 of 536 aneurysms (72.2%, 95% CI: 68.4%–76.0%) at 12 months. Complications were reported in all 13 studies. Short-term complications occurred in 94 (12.7%, 95% CI: 10.3%–15.0%) and intermediate complications in 42 (5.7%, 95% CI: 4.0%–7.3%) patients. Of the total complications, 110 (14.8%, 95% CI: 12.3%–17.4%) were classified as hypoperfusion and 9 (1.2%, 95% CI: 0.4%–2.0%) as haemorrhagic. Six of these complications were intracranial haematomas and three were related to the groyne arterial access. The additional 17 complications (2.3%, 95% CI: 1.2%–3.4%) were classified as ‘other’, including four that were either ischaemic or haemorrhagic but could not be definitively assigned to either category based on the available data. The overall number of complications for all 743 patients at 12 months was 136 (18.3%, 95% CI: 15.5%–21.1%). Long-term clinical outcomes at 12 months based on individual mRS scores were available for 102 patients, of which 11 had mRS ⩾1 (10.8%, 95% CI: 4.8%–16.8%), 3 mRS ⩾2 (2.9%, 95% CI: 0.0%–6.2%) and 2 mRS ⩾3 (2.0%, 95% CI: 0.0%–4.7%), including one reported death. Another study only reported on dichotomised long-term outcome with mRS ⩾3 in four of 126 patients (3.2%, 95% CI: 0.1%–6.2%, including two reported deaths).

## Discussion

Our systematic review identified only uncontrolled patient series with poor or at highest moderate methodological quality on FD-stent treatment of saccular, non-giant UIA that often can also be treated with regular coiling or clipping. Apart from insufficient methodological quality, half of these studies are also at risk of bias because of financial conflicts of interest of authors. The available data suggest that one in six patients had a complication, leading to some kind of disability in individual cases. Because most of the included series were retrospective and none had a good methodological quality, these proportions are likely to be an underestimation. Moreover, in one in four patients the aneurysm was not occluded at 1-year follow-up.

Our findings on conflicts of interest are consistent with those of a recent systematic review, examining all conflicts of interests in the 269 scientific articles published and printed in 2021 in the journal of neurointerventional surgery. In this review, 42% of articles disclosed at least one conflict of interest, and 92% of these articles contained relevant financial conflicts, defined similarly as we did in our study.^28^

This review included predominantly studies with small anterior circulation UIAs, for which established treatments such as coiling or clipping are available and FDA-approval for FD-stent treatment is often missing. In a randomised trial comparing clipping and endovascular treatment, poor outcome defined as mRS >2 occurred in 2%–3% of patients.^4^ New neurological deficits occurred in 22% after surgical and 12% following endovascular treatment, highlighting that also established occlusion methods carry risk for complications. A direct comparison between those data and the pooled data in our review is inappropriate, also because of the difference in methodological quality between the randomised trial and the observational, mostly retrospective studies underlying our pooled analysis. However, the proportions of patients with anterior circulation aneurysms and small aneurysms in the trial and in our review are comparable and therefore do not suggest that the aneurysms in the studies included in our review are at higher risk of complications. In another randomised controlled trial, comparing FD-stent treatment with standard treatment (coiling or clipping) for both ruptured and unruptured intracranial aneurysms, the proportion of patients with mRS >2 was 7% after FD-stent treatment. However, over 10% of the aneurysms in this study were classified as giant and approximately 40% were larger than 10 mm, which implies that the aneurysms included were complex and may have not always be amenable to safe coiling or clipping.^29^ A recent meta-analysis reported an ischaemic complication rate of 16% after FD-stent treatment, which is comparable to our observations.^30^ This might in part explain poor neurological outcomes and is observed more frequently compared to clipping and coiling.^5^ Another meta-analysis of long-term outcomes following FD-stent treatment in UIA reported a complete occlusion rate of 77% at 1-year post-treatment, which is consistent with our findings.^11^ In comparison, occlusion rates are higher after coiling and clipping.^31,32^

The high rate of patients with post-treatment morbidity (mRS >0) can in part be explained by pre-treatment mRS scores, although this information was only available for 67% of the patients. In particular, one of the largest studies in our meta-analysis used mRS 0–2 as its definition of favourable outcome, which precluded the detection of mRS shifts from 0 to 1 or 2, or from 1 to 2.^20^ In addition, our restriction to studies reporting also short-term clinical outcomes within 1–3 months resulted in exclusion of studies with larger patient populations. However, we purposely included studies reporting also short-term outcomes to capture the rate of immediate complications from aneurysm treatment. The poor to moderate methodological quality of the included studies, the presence of financial conflicts of interest in more than half and the uncertainty about unbiased outcome assessment in two-thirds the studies limit the clinical validity of the available data.

This analysis has several strengths. First, in contrast to other studies that often neglected the risk of short-term complications, we evaluated short-term clinical outcomes after FD-stent treatment. Moreover, we focussed on non-giant and non-blister UIAs, for which often established treatment options are available. Thus, we aimed to provide an assessment of the potential role of FD-stents as a viable alternative in routine UIA management. Third, by systematically assessing study quality and conflicts of interest, potential biases could be related to each study and thereby putting the clinical results into a more objective context. Our study also has limitations. Differences in outcome definitions and baseline characteristics precluded pooling of existing data of all studies in some analyses and pooling was performed without a random-effects model which may have led to over- or underestimation of the proportions. Second, although we used three databases with specific search terms, we still may have missed relevant studies. Third, no study protocol has been registered or published beforehand. Finally, due to the low methodological quality of the underlying studies and the risk of bias by financial conflicts of interest, it was inherently not possible to draw definitive conclusions about whether FD-stents were superior or inferior to conventional treatments.

## Conclusions

The available data on FD-stent treatment for small, anterior circulation UIAs show that studies are methodologically weak, subject to potential bias by financial interests and suggest that FD-stent treatment is associated with relevant proportions of complications and post-treatment morbidity. Before more robust guideline recommendations on FD-stent treatment for UIAs can be formulated, unbiased good-quality RCTs are needed that compare safety (short-term clinical outcome and incidence of steno-occlusive complications), efficacy (degree of UIA occlusion) and durability (long-term protection against aneurysmal rupture) between FD-stent treatment and standard treatment such as coiling or microsurgical clipping.

## Data Availability

Not applicable.
